# Health care professionals’ attitudes towards youth-friendly sexual and reproductive health services in Jordan: a cross-sectional study of physicians, midwives and nurses

**DOI:** 10.1186/s12978-021-01137-4

**Published:** 2021-04-21

**Authors:** Jewel Gausman, Areej Othman, Raeda Al-Qotob, Abeer Shaheen, Eman Abu Sabbah, Mohannad Aldiqs, Iqbal Hamad, Maysoon Dabobe, Ana Langer

**Affiliations:** 1 Department of Global Health and Population, Women & Health Initiative, Harvard TH Chan School of Public Health, 677 Huntington Ave, Boston, MA 02115 USA; 2 Department of Maternal and Child Health Nursing, School of Nursing, University of Jordan, Queen Rania Street, Amman, Jordan; 3 Department of Family and Community Medicine, School of Medicine, University of Jordan, Queen Rania Street, Amman, Jordan; 4 Department of Community Health Nursing, School of Nursing, University of Jordan, Queen Rania Street, Amman, Jordan; 5 School of Nursing, Al-Ahliyya Amman University, Amman, Jordan; 6 Jordanian Hashemite Fund for Human Development, 127 Madina Street, Amman, Jordan

**Keywords:** Adolescents, Sexual and reproductive health, Youth-friendly, Health services, Youth, Jordan, Attitudes

## Abstract

**Background:**

Youth-friendly sexual and reproductive health (SRH) services are thought to make such services for adolescents more accessible and acceptable; however, provider attitudes may still present an important barrier. Improving youth SRH service utilization has been recognized as a national priority in Jordan; however, existing services remain underutilized. Previous studies found that youth perceive SRH services to be inadequate and that providers are not supportive of their needs. The purpose of this study is measure provider attitudes towards youth-friendly SRH services and explore their variation according to individual characteristics among health care professionals in Jordan.

**Methods:**

We measured provider attitudes towards youth-friendly SRH services using a scale that was developed and validated in Jordan. The scale consists of three subscales: (1) Attitudes towards SRH information and services offered to youth, (2) Norms and personal beliefs, and (3) Attitudes towards the policy and clinical environment. Possible scores range between 1 and 4, with higher scores reflecting more youth-friendly attitudes. Physicians, midwives and nurses working at either primary health centers, comprehensive care centers, or women’s and children’s health centers where services to adolescents are or should be offered were recruited from four governorates in Jordan using a two-stage, cluster sampling scheme. Differences in attitudes were assessed using simple and multivariable linear regression analysis.

**Results:**

The sample consisted of 510 providers from four governorates in Jordan. The mean provider score on the full scale was 2.7, with a range of 2.0 to 3.8. On Subscales 1 and 2, physicians exhibited significantly more youth-friendly attitudes than nurses by scoring 0.17 points higher than nurses on Subscale 1 (95% CI: 0.02–0.32; p < 0.05) in adjusted analyses. Providers who had been previously trained in SRH issues scored 0.10 points higher (95% CI: 0.00—0.20; p < 0.05) than those who had not on Subscale 3. No differences were found according to provider characteristics on Subscale 2. Providers exhibited the lowest scores related to items referencing youth sexual behavior.

**Conclusions:**

Provider attitudes towards youth-friendly SRH service delivery highlight context-specific, cultural concerns. The limited variation in attitudes related to norms and personal beliefs may be a reflection that such beliefs are deeply held across Jordanian society. Last, as past training on SRH was significantly associated with higher scores, our results suggest opportunity for intervention to improve providers’ confidence and knowledge.

## Plain Language Summary

Making sexual and reproductive (SRH) services easier for youth to access, organizing service delivery in a way that meets youth’s needs, and supporting health care professionals to interact with youth in a friendly manner can make SRH services more youth-friendly. If SRH services are more youth-friendly, more youth may use them. In Jordan, steps have been taken to make SRH services more youth-friendly, but youth still do not believe that providers are supportive of their needs. This study aims to measure physician’s, nurse’s, and midwives’ attitudes towards youth-friendly SRH services in Jordan. We also look at whether certain individual characteristics, such as age, type of service provider, etc. are related to provider attitudes We used a scale that tested in Jordan to measure provider attitudes. The scale focuses on three domains: (1) Attitudes towards SRH information and services offered to youth, (2) Norms and personal beliefs, and (3) Attitudes towards the policy and clinical environment. Possible scores range between 1 and 4, with higher scores reflecting more youth-friendly attitudes. Our sample includes 510 health care providers from four regions in Jordan. We used descriptive statistics and regression analysis to conduct our analysis. Our results show that physicians had more supportive attitudes than nurses or midwives on Subscales 1 and 3. Providers who reported having been trained in SRH issues in the past had higher scores on Subscale 3. No individual characteristics were related to Subscale 2. We find that in Jordan, provider attitudes may reflect deeply rooted cultural norms.

## Background

Across the Middle East and much of the world, youth face considerable barriers when trying to access sexual and reproductive health (SRH) services. Many of the barriers that prevent youth from obtaining SRH services originate from within the service delivery environment [1, 2]. Health service providers may exhibit unfriendly or judgmental attitudes towards youth, behave in a way that is disrespectful or stigmatizing, provide youth with poor quality of care, or unnecessarily restrict access to certain services to youth by requiring parental or spousal permission when not required [3–5]. Further, there may not be clear service guidelines on what services should be provided to youth, thus, leaving providers to offer services based on their own discretion or their own personal values and beliefs [4–8].

Offering youth-friendly SRH services is thought to improve service utilization among youth [8, 9]. There are several definitions for what constitute youth-friendly SRH services. In general, youth-friendly services are those that attract youth, provide a comfortable setting for youth, meet the specific service delivery needs of youth, and retain their youth clientele [10]. Other definitions focus on ensuring that youth-friendly services are accessible, acceptable, equitable, appropriate, and effective [11]. Previous research has found that provider attitudes towards youth are among the most important factors in shaping youth’s perceptions of whether SRH services are youth-friendly, and they are associated with utilization of SRH services by youth [12–14]. Globally, few studies have sought to measure provider attitudes within the context of providing youth-friendly services in a systematic way using validated tools.

Improving SRH service utilization among youth has been identified as a national priority in Jordan [15]; however, there remains no clear definition of how this priority translates into changes at the service delivery level [16]. Further, existing SRH services continue to remain largely underutilized by youth in Jordan. The Ministry of Health estimates that only 1% of adolescents access primary health care [17], and there are no available statistics on SRH service utilization within this population. Youth-friendly services have been introduced in a small number of health centers that target women and children throughout the country, although implementation is not systematic and there are no clear criteria as to what makes services “youth-friendly” [18]. In general, a systematic landscape analysis of initiatives to improve SRH service delivery reveals that there has been very little programmatic investment in this domain to date [16].

Previous research in Jordan has identified several challenges that relate to provider–client interactions, especially among youth, that may negatively influence SRH service provision. In particular, SRH services provided to youth have been found to be of poor quality [17] and highlight provider misconceptions about appropriate service provision to youth. For example, several studies have found that youth are often advised not to use contraception because of fears over side effects including infertility [19–22]. Further, given the strict social and religious norms that prohibit sexual activity among youth who are unmarried, youth in Jordan have expressed concerns related to privacy and confidentiality within the clinic setting [23, 24]. Other studies highlight youth’s concerns specifically relating to provider attitudes during provider–client interaction. Youth indicate that they find SRH services unpleasant, inadequate, and unprofessional, and they believed that providers do not take them seriously, do not know what information they need, and view their questions as inappropriate [25, 26].

Globally, and in Jordan, there has been limited research designed to understand health professionals’ perspectives in providing SRH services towards youth. To help fill this gap, the aim of this study was to describe health professionals’ attitudes towards the provision of youth-friendly SRH services using a tool that was developed and validated in Jordan [27]. Further, we examined differences in provider attitudes related to their demographic characteristics, background experience and training, and provider type. The results of this study will help support the development of youth-friendly services across Jordan, while providing important insight relevant to programs across the Middle East.

## Methodology

### Study design

This is a cross-sectional study conducted in health facilities in four governorates in Jordan: Amman, Irbid, Mafraq, and Zarqa. This study was part of a larger study focused on adolescent SRH amongst Jordanian and Syrian youth, and as such, the four governorates were selected purposively due to their large population of Syrian refugees. Ethical approval was obtained from the Institutional Review Boards at the Harvard T.H. Chan School of Public Health and the University of Jordan. We used the STROBE cross-sectional checklist when writing the report of this study [28].

### Setting

Data collection occurred in primary health centers, comprehensive care centers, and women’s and children’s health centers.

### Study population

The study population includes all primary care physicians, midwives, and nurses employed in health facilities in Amman, Irbid, Mafraq, or Zarqa governorates.

### Participant selection

This study used a two-stage sampling scheme. In the first stage, health facilities were randomly selected from amongst a facility listing obtained from the Ministry of Health including all public health facilities in each of the four governorates. In the second stage, participants were recruited from amongst the study population by convenience. All primary care physicians, midwives and nurses physically present at the facility at the time of recruitment were eligible to participate.

We used the following equation to calculate the per-governorate sample size that incorporates the design effect to account for clustering in the data by facility:

$$N={((t}^{2}*p(1-p))/m^2)*{D}_{eff}$$

where: N = required sample, t = value of confidence level, p = estimated prevalence of the indicator of interest, m = margin of error, D_eff_ = intra-cluster correlation.

Based on the equation above, using a 95% CI and a margin of error of 0.1, we assumed that the prevalence (p) of youth-friendly attitudes was 0.5 in order to maximize the required sample size, as the true value is unknown. Further, we assumed an intra-cluster correlation within facilities of 1.2 [29]. We arrived at a required sample size of 116 per governorate, and a total minimum sample of 464 providers needed for the study. Based on facility staffing patterns, we estimated that 20 facilities from each governorate would need to be included in our random sample to reach our required sample size.

### Data collection

Data were collected by pen and paper survey in order to limit social desirability bias given the sensitivity of the topic in this setting.

#### Dependent variable: provider attitudes

To measure provider attitudes towards the provision of youth-friendly services, we used a scale that was developed and validated in a pilot study of health service providers in Amman, Jordan to assess the scale’s psychometric properties using factor analysis[27]. The scale was found to have high internal consistency reliability, with Cronbach’s alpha estimated at 0.84. The overall scale includes three subscales: 1) Attitudes towards SRH information and services offered to youth, 2) Norms and personal beliefs, and 3) Attitudes towards the clinical and policy delivery environment. The scale focuses on operationalizing the World Health Organization’s definition of youth-friendly services from a quality of care perspective, highlighting the concepts of accessibility, acceptability, equity, appropriateness, and effectiveness [11]. The items in the scale focus on a range of topics of importance to Jordan and the Middle East, such as sexual and gender-based violence, youth sexuality, and contraceptive use. Responses to each item in the scale ranged from 1 (“strongly disagree”) to 4 (“strongly agree”). In order to reduce bias, surveys were administered by pen and paper in a private room and included other questions on covariates of interest, including questions related to provider demographics, background and experience.

#### Independent variables

Respondents were asked to complete a brief survey containing several demographic questions, including provider type, age, sex, marital status, religion, location, years of experience working as a health care provider, and if they had ever received training on SRH issues.

#### Data analysis

We first calculated descriptive statistics on the overall sample, as well as for each item, subscale, and the full scale. To improve interpretability, negative items were reverse-coded and scale scores were normalized across both the full scale and the three subscales to reflect a range of 1 to 4, with lower scores reflecting less youth-friendly attitudes and higher scores reflecting more youth-friendly attitudes. We excluded participants with more than three missing responses on scale items (n = 19). To ensure that the overall scale exhibited high internal consistency reliability, we calculated Cronbach’s alpha using our sample. We explored variation in responses by providers’ characteristics using simple linear regression analysis. Further, we examined the associations between providers’ attitudes as measured by the overall scale and each subscale using multivariable adjusted linear regression, including providers’ age, sex, location, provider type, and whether the provider reported having had training on SRH issues.

## Results

The initial sample included 529 participants; however, 19 were excluded due to missing data. The final sample consisted of 510 providers from four governorates in Jordan: Amman (33.7%), Irbid (22.1%), Mafraq (23.4%) and Zarqa (20.8%). Table 1 describes overall sample characteristics. The majority of providers were between the ages of 25–35 years (48.5%), were female (81.3%), and Muslim (98.7%). Nurses (41.2%) and midwives (38.7%) constituted the majority of the sample; 19% of respondents were primary care physicians. More than 80% of the sample had less than 10 years of experience in practice and 61% reported having received training on SRH issues. Overall, the scale exhibited high internal consistency reliability. Cronbach’s alpha for the full scale was estimated at 0.71, which is generally considered to be good [30].

**Table 1:** Sample characteristics

Provider characteristics	No	%
Total	529	100
Age		
18–24 years	46	8.7
25–35 years	257	48.58
36–45 years	154	29.11
46 + years	70	13.23
Missing	2	0.38
Sex		
Male	99	18.71
Female	430	81.29
Marital status		
Married	420	79.4
Unmarried	104	19.66
Missing	5	0.95
Religion		
Muslim	522	98.68
Christian	7	1.32
Governorate		
Amman	178	33.65
Irbid	117	22.12
Mafraq	124	23.44
Zarqa	110	20.79
Location type		
Rural	192	36.29
Urban	328	62
Missing	9	1.6
Provider type		
Midwife	204	38.56
Nurse	218	41.21
Primary care physician	101	19.09
Missing	8	1.14
Years of experience		
Less than 5	244	46.12
5–10 years	192	36.29
11–20 years	16	3.02
20 + years	53	10.02
Missing	24	4.54
Ever received training on sexual and reproductive health		
Yes	321	60.68
No	201	38
Missing	7	1.32

**Fig. 1:**
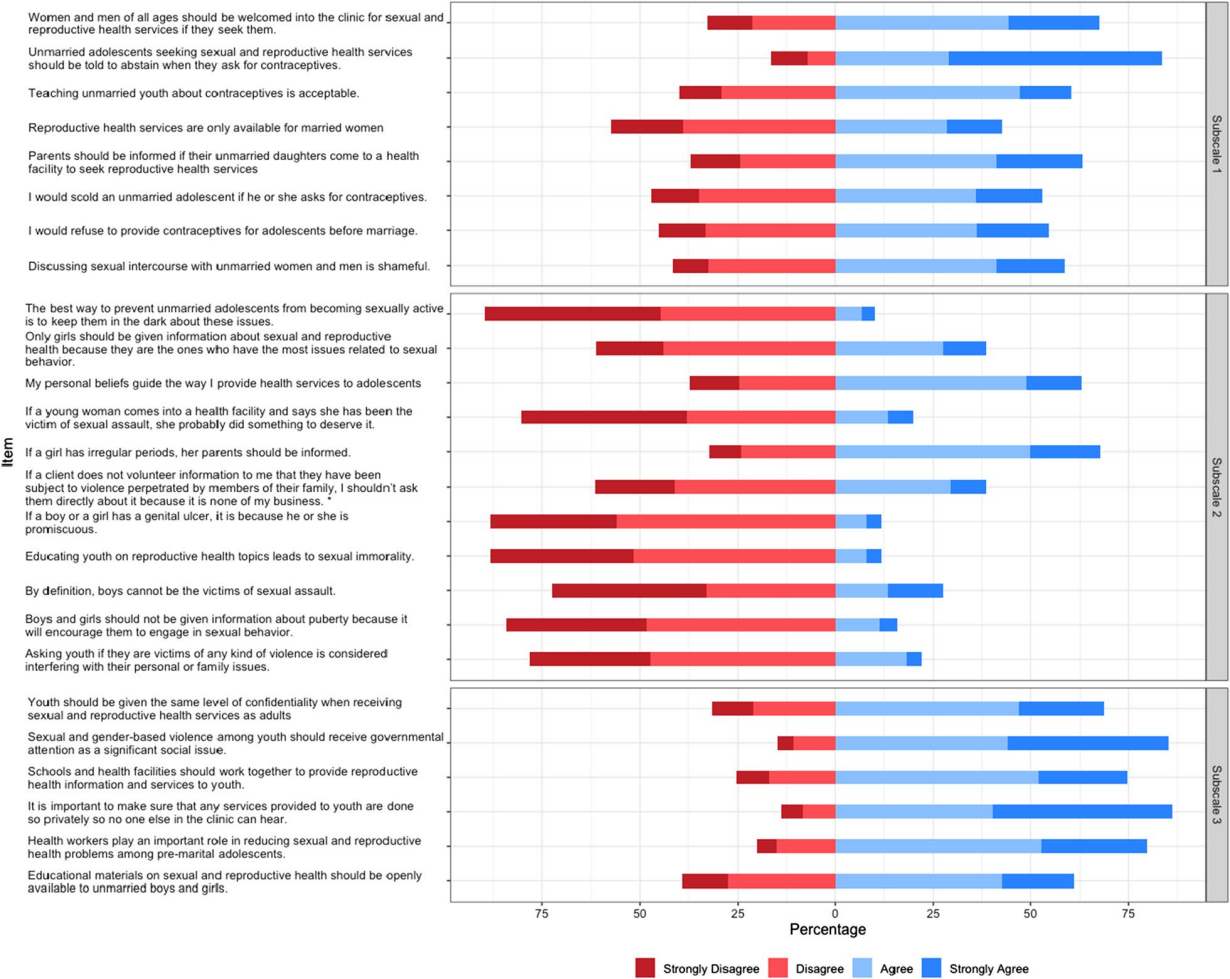
Provider responses to individual scale items measuring attitudes towards youth-friendly SRH service

The mean provider score on the full scale was 2.7, with a range of 2.0 to 3.8. Raw responses for each item are presented in Fig. 1. Table 2 presents the mean normalized scores for each subscale and each individual scale item. In general, providers scored highest on Subscale 3: “Attitudes towards the policy and clinical environment,” for which the mean provider score was estimated at 3.0, and lowest on Subscale 1: “Attitudes towards information and services offered to youth,” for which the mean provider score was estimated at 2.4.

**Table 2:** Mean item and subscale scores measuring provider attitudes towards youth-friendly sexual and reproductive health services, scored from 1–4 points (1 = unfavorable attitudes, 4 = most supportive attitudes), n = 529

Subscale 1: Attitudes towards information and services offered to youth	Mean (SD)
*Subscale 1 Combined Score*	*2.4 (0.46)*
Unmarried adolescents seeking sexual and reproductive health services should be told to abstain when they ask for contraceptives^a^	1.7 (0.95)
Discussing sexual intercourse with unmarried women and men is shameful^a^	2.3 (0.87)
Unmarried adolescents should not be provided with contraceptives because culture and religion prohibit engagement in premarital sex^a^	2.3 (1.0)
Parents should be informed if their unmarried daughters come to a health facility to seek reproductive health services^a^	2.3 (0.94)
I would scold an unmarried adolescent if he or she asks for contraceptives^a^	2.4 (0.91)
I would refuse to provide contraceptives for adolescents before marriage^a^	2.4 (0.92)
Teaching unmarried youth about contraceptives is acceptable	2.6 (0.84)
Reproductive health services are only available for married women^a^	2.6 (0.96)
Women and men of all ages should be welcomed into the clinic for sexual and reproductive health services if they seek them	2.8 (0.92)
*Subscale 2: Norms and personal beliefs*	*Mean (SD)*
*Subscale 2 Combined Score*	*2.9 (0.39)*
If a girl has irregular periods, her parents should be informed^a^	2.2 (0.83)
My personal beliefs guide the way I provide health services to adolescents^a^	2.4 (0.87)
Only girls should be given information about sexual and reproductive health because they are the ones who have the most issues related to sexual behavior^a^	2.7 (0.89)
If a client does not volunteer information to me that they have been subject to violence perpetrated by members of their family, I shouldn’t ask them directly about it because it is none of my business^a^	2.7 (0.89)
By definition, boys cannot be the victims of sexual assault^a^	3.0 (1.0)
Asking youth if they are victims of any kind of violence is considered interfering with their personal or family issues^a^	3.1 (0.81)
Boys and girls should not be given information about puberty because it will encourage them to engage in sexual behavior^a^	3.2 (0.78)
Educating youth on reproductive health topics leads to sexual immorality^a^	3.2 (0.75)
If a boy or a girl has a genital ulcer, it is because he or she is promiscuous^a^	3.2 (0.72)
If a young woman comes into a health facility and says she has been the victim of sexual assault, she probably did something to deserve it.*	3.2 (0.88)
The best way to prevent unmarried adolescents from becoming sexually active is to keep them in the dark about these issues^a^	3.3 (0.74)
*Attitudes towards the policy and clinical environment*	Mean (SD)
*Subscale 3 Combined Score*	3.0 (0.53)
Educational materials on sexual and reproductive health should be openly available to unmarried boys and girls	2.7 (0.90)
Youth should be given the same level of confidentiality when receiving sexual and reproductive health services as adults	2.8 (0.90)
Schools and health facilities should work together to provide reproductive health information and services to youth	2.9 (0.85)
Health workers play an important role in reducing sexual and reproductive health problems among pre-marital adolescents	3.0 (0.79)
Sexual and gender-based violence among youth should receive governmental attention as a significant social issue	3.2 (0.79)
It is important to make sure that any services provided to youth are done so privately so no one else in the clinic can hear	3.3 (0.84)

^a^Negatively worded items were reverse-coded so that a higher score reflects more positive attitudes towards youth-friendly services

For Subscale 1, “Attitudes towards information and services offered to youth,” the means for each individual item presented in Table 2 suggest that providers exhibited the most unfavorable attitudes towards items that pertain specifically to sexual activity among unmarried youth and issues related to informing parents. In Subscale 1, providers scores suggest the least youth-friendly attitudes on items such as “Unmarried adolescents seeking sexual and reproductive health services should be told to abstain when they ask for contraceptives” (mean = 1.7), “Discussing sexual intercourse with unmarried women and men is shameful” (mean = 2.3), and “Parents should be informed if their unmarried daughters come to a health facility to seek reproductive health services” (mean = 2.3). The two items in Subscale 1 that focus on contraceptive provision to unmarried adolescents also exhibited relatively low mean scores (mean = 2.4), specifically, these items focused on refusal to provide services and scolding youth for wanting to obtain contraceptives. On this scale, provider attitudes were more favorable towards items that reference reproductive health services in a more general way, or those that reference education about contraceptives, than those that directly reference sexual activity or provision of contraceptives.

For Subscale 2, “Norms and personal beliefs,” the item on which providers exhibited the least youth-friendly attitudes was “If a girl has irregular periods, her parents should be informed” (mean = 2.2). Additionally, providers scored relatively low on the item “My personal beliefs guide the way I provide health services to adolescents” (mean = 2.4). The items with the highest average scores include those related to normative statements about whether educating youth leads to sexual activity, such as “Educating youth on reproductive health topics leads to sexual immorality” (mean = 3.2) and those related to making value-based assumptions about youth should they seek certain services, such as “If a boy or a girl has a genital ulcer, it is because he or she is promiscuous” (mean = 3.2) and “If a young woman comes into a health facility and says she has been the victim of sexual assault, she probably did something to deserve it” (mean = 3.2).

In Subscale 3, while scores for individual items were generally higher than those found in the other subscales, the lowest scoring item pertained specifically to unmarried youth: “Educational materials on sexual and reproductive health should be openly available to unmarried boys and girls” (mean = 2.7). The second lowest item pertained to confidentiality of services specifically towards youth “Youth should be given the same level of confidentiality when receiving sexual and reproductive health services as adults” (mean = 2.8). In contrast, the highest scoring item pertained to a specific dimension of confidentiality in services delivered to youth “It is important to make sure that any services provided to youth are done so privately so no one else in the clinic can hear” (mean = 3.3).

Table 3 presents scale means according to provider characteristics as well as the results of the simple linear regression analysis of attitude scores on provider characteristics for each subscale and the full scale. Results suggest that certain provider characteristics are associated with provider attitudes as measured by each subscale individually and the full scale. The results highlight significant geographic variation according to governorate, by which providers in Amman have the most favorable attitudes towards youth-friendly services with regard to Subscale 1 and 3. The results for subscale 1 indicate that providers in Irbid and Zarqa score on average − 0.15 (− 0.27 to − 0.05; p < 0.01) and − 0.18 (− 0.29 to − 0.07; p < 0.001) points lower than providers in Amman, respectively. Similarly, on Subscale 3, providers in Irbid and Mafraq score − 0.22 (− 0.33 to − 0.90; p < 0.001) and − 0.18 (− 0.30 to − 0.06; p < 0.05) points lower than providers in Amman, respectively. Results also suggest that primary care physicians have more favorable attitudes towards the provision of youth-friendly services according to Subscale 1 and Subscale 3 when compared to other types of providers, with scores that are on average 0.21 (0.10–0.32; p < 0.001) and 0.19 (0.06–0.31; p < 0.01) points higher than nurses, respectively. There were no significant differences apparent in attitude score between nurses and midwives. Female providers showed consistently lower attitudes when compared to males in Subscale 1 and Subscale 3. Female providers scored 0.16 (− 0.26 to − 0.06; p < 0.01) and − 0.12 (− 0.23 to − 0.09; p < 0.05) points lower on Subscale 1 and Subscale 3, respectively. Past provider training on SRH was associated with more favorable attitudes on the full scale, with providers having had training scoring 0.6 (0.01–0.12; p < 0.05) points higher than those who received no training. No significant differences in provider attitudes were observed according to provider marital status, religion, whether they practiced in a rural or urban area, years of work experience, or facility type. No provider characteristics were found to be associated with scores on Subscale 2.

**Table 3:** Mean scores and univariate linear regression analysis of provider attitudes towards youth-friendly sexual and reproductive health services according to provider characteristics (n = 510)

	Subscale 1		Subscale 2		Subscale 3		Full scale	
	Mean (SD)	Simple OLS	Mean (SD)	Simple OLS	Mean (SD)	Simple OLS	Mean (SD)	Simple OLS
		Coefficient (95% CI)		Coefficient (95% CI)		Coefficient (95% CI)		Coefficient (95% CI)
Age								
18–24 years (ref)		1	2.82 (0.34)	1	2.50 (0.35)	1	2.66 (0.25)	1
25–35 years	2.39 (0.46)	0.13 (− 0.13, 0.28)	2.92 (0.42)	0.11 (− 0.19, 0.23)	3.01 (0.54)	0.06 (− 0.13, 0.22)	2.76 (0.32)	0.11 (0.13, 0.20)
36–45 years	2.36 (0.44)	0.09 (− 0.06, 0.25)	2.91 (0.37)	0.09 (− 0.04, 0.22)	2.91 (0.54)	− 0.04 (− 0.21, 0.14)	2.72 (0.27)	0.07 (− 0.03, 0.16)
46 + years	2.50 (0.47)	0.23 (0.06, 0.41) **	2.90 (0.40)	0.08 (− 0.07, 0.23)	2.98 (0.59)	0.04 (− 0.16, 0.23)	2.78 (9.28)	0.12 (0.02, 0.24)*
Sex								
Male	2.5 (0.42)	1	2.89 (0.43)	1	3.08 (0.50)	1	2.80 (0.29)	1
Female	2.36 (0.46)	− 0.16 (− 0.26, − 0.06)**	2.91 (0.39)	0.25 (− 0.06, 0.11)	2.95 (0.53)	− 0.12 (− 0.23, − 0.01)*	2.73 (0.29)	− 0.07 (− 0.13, − 0.01)*
Governorate								
Amman	2.45 (0.50)	1	2.96 (0.41)	1	3.08 (0.53)	1	2.81 (0.33)	1
Irbid	2.30 (0.46)	− 0.15 (− 0.27, − 0.05)**	2.89 (0.41)	− 0.07 (− 0.16, 0.24)	2.87 (0.62)	− 0.22 (− 0.33, − 0.09)***	2.68 (0.28)	− 0.13 (− 0.20, − 0.06)***
Mafraq	2.46 (0.40)	0.01 (− 0.10, 0.11)	2.88 (0.38)	− 0.08 (− 0.17, 0.01)	2.90 (0.46)	− 0.18 (− 0.30, − 0.06)**	2.74 (0.27)	− 0.07 (− 0.14, − 0.01)*
Zarqa	2.27 (0.42)	− 0.18 (− 0.29, − 0.07)***	2.91 (0.39)	− 0.05 (− 0.14, 0.45)	3.01 (0.43)	− 0.08 (− 0.20, 0.05)	2.71 (0.25)	− 0.10 (-0.17, − 0.33)**
Provider type								
Midwife	2.37 (0.46)	1	2.89 (0.37)	1	2.94 (0.53)	1	2.72 (0.29)	1
Nurse	2.32 (0.45)	− 0.05 (− 0.14, 0.37)	2.90 (0.43)	0.01 (− 0.07, 0.08)	2.93 (0.53)	0.00 (− 0.10, 0.1)	2.71 (0.28)	− 0.01 (− 0.07, 0.44)
Primary care physician	2.58 (0.43)	0.21 (0.10, 0.32)***	2.94 (0.44)	0.05 (− 0.05, 0.14)	3.12 (0.51)	0.19 (0.06, 0.31)**	2.85 (0.322)	0.14 (0.07, 0.21)***
Ever received training on SRH								
No	2.35 (0.45)	1	2.87 (0.41)	1	2.78 (0.45)	1	2.71 (0.29)	1
Yes	2.41 (0.47)	0.05 (− 0.3, 0.14)	2.93 (0.39)	0.06 (− 0.01, 0.13)	3.01 (0.54)	0.08 (− 0.23, 0.18)	2.76 (0.276)	0.6 (0.01, 0.12)*

*p < 0.05; **p < 0.01; ***p < 0.001

Table 4 presents the results of the multivariable adjusted regression analysis for each subscale and the full scale. As in the unadjusted analyses, provider characteristics remained significantly associated with attitudes scores. Type of provider was associated with scores for Subscale 1, Subscale 3 and the full scale. No significant differences were found in attitudes between nurses and midwives with regard to any of the scales; however, primary care physicians exhibited significantly more youth-friendly attitudes by scoring 0.17 points higher than nurses on Subscale 1 (95% CI: 0.02–0.32; p < 0.05), 0.17 points higher than nurses on Subscale 3 (95% CI: 0.00–0.35; p < 0.05), and 0.14 points higher than nurses on the full scale (95% CI: 0.05–0.24; p < 0.01). Adjusted models also suggest that there remains significant regional variation in scores. In general, providers in Amman exhibited the most youth-friendly attitudes on Subscales 1 and 3, and the full scale, with providers in Irbid and Zarqa exhibiting significantly less youth-friendly attitudes in Irbid for Subscale 1 and providers in Irbid and Mafraq exhibiting significantly less youth-friendly attitudes for Subscale 3. Last, in fully adjusted models, provider training was associated with improved attitudes only on Subscale 3, so that providers who have been trained on SRH issues scored 0.10 points higher (95% CI: 0.00—0.20; p < 0.05).

**Table 4:** Multivariable adjusted linear regression analysis of provider attitudes towards youth-friendly sexual and reproductive health services on provider characteristics (n = 510)

	Subscale 1	Subscale 2	Subscale 3	Full scale
	OLS coefficient (95% CI)	OLS coefficient (95% CI)	OLS coefficient (95% CI)	OLS coefficient (95% CI)
Age				
18–24 years	1	1	1	1
25–35 years	0.04 (− 0.13. 0.20)	0.12 (− 0.4, 0.26)	− 0.04 (− 0.15, 0.23)	− 0.07 (− 0.04, 0.17)
36–45 years	0.04 (− 0.14, 0.22)	0.10 (− 0.06, 0.26)	− 0.04 (− 0.25, 0.17)	0.05 (− 0.07, 0.16)
46 + years	0.08 (− 0.12, 0.28)	0.08 (− 0.10, 0.26)	− 0.05 (− 0.28, 0.18)	0.05 (− 0.08, 0.18)
Sex				
Male	1	1	1	1
Female	− 0.02 (− 0.16, 0.11)	0.01 (− 0.02, 0.22)	− 0.3 (− 0.19, 0.13)	0.03 (− 0.06, 0.11)
Governorate				
Amman	1	1	1	1
Irbid	− 0.15 (− 0.26, − 0.04)**	− 0.07 (− 0.17, 0.03)	− 0.19 (− 0.31, − 0.06)**	− 0.12 (− 0.19, − 0.05)***
Mafraq	0.01 (− 0.08, 0.12)	− 0.07 (− 0.17, 0.03)	0.18 (− 0.30, − 0.05)**	− 0.06 (− 0.13, 0.00)
Zarqa	− 0.15 (− 0.27, − 0.03)*	− 0.01 (− 0.11, 0.09)	− 0.05 (− 0.19, 0.08)	− 0.07 (− 0.14, 0.01)
Provider type				
Midwife	1	1	1	1
Nurse	− 0.07 (− 0.17, 0.03)	0.01 (− 0.08, 0.09)	0.01 (− 0.10, 0.12)	− 0.01 (− 0.08, 0.05)
Primary Care Physician	0.17 (0.02, 0.32)*	0.10 (− 0.03, 0.23)	0.17 (0.00, 0.35)*	0.14 (0.05, 0.24)**
Ever received training on SRH				
No	1	1	1	1
Yes	0.04 (− 0.05, 0.12)	0.04 (− 0.03, 0.12)	0.10 (0.00, 0.20)*	0.05 (0.00, 0.11)

^*^p < 0.05; **p < 0.01; ***p < 0.001

## Discussion

The results of this study highlight several important issues with regard to provider attitudes towards youth-friendly service provision in Jordan that are relevant to both programs and policies across the Middle East. First, provider attitudes towards youth-friendly SRH service delivery highlight several context-specific cultural concerns. Second, the results suggest that provider attitudes related to norms and personal beliefs, as measured by Subscale 2, appear to be the most static of the three domains investigated, and are generally invariant in relation to individual characteristics. Last, as past training is significantly associated with attitudes towards the policy and clinical environment, our results suggest opportunity for intervention within this domain.

Our results reinforce the important role that cultural concerns play in influencing provider attitudes towards the provision of youth-friendly SRH services. In our study, providers appeared to have the least favorable attitudes towards issues that relate specifically to youth sexuality, especially among unmarried youth. In Jordan, religious principles prohibit sexual relationships outside of marriage and the discussion of sexuality is generally considered to be taboo according to traditional norms [31]. The results of our study suggest that providers attitudes are influenced by these same traditional norms in a way that stigmatizes youth sexuality, despite the provider’s responsibility to offer care. Other studies focusing on adolescent SRH services have found that providers often find themselves facing conflict between their own personal values, dominant norms in the community, and their role as caregivers [32, 33]. Furthermore, the results of our study are similar to previous research conducted in relatively conservative, religious settings, where providers often exhibit conflicting perspectives in relation to adolescent sexuality. On one hand, providers may disapprove of premarital sexual activity among adolescents, but they may also simultaneously exhibit a pragmatic attitude by approving of the use of contraception by adolescents who are sexually active [4]. This may help to explain the seemingly contradictory results of our study in relation to the items that mention contraceptives. In Jordanian society, where norms restrict sexual activity to marriage, the use of contraception to prevent pregnancy is also limited to marriage. While providers tend to display less favorable attitudes towards items that mention contraceptive use among youth within the context of sexual activity outside of marriage, they are comparatively more supportive of items that focus on contraception in general, as they may also consider contraceptive use for other medical indications that do not relate to unsanctioned sexual activity.

In a similar light, our results also highlight a similar issue with respect to different aspects of confidentiality. Providers generally scored fairly low on items that pertain to informing parents if their children seek SRH services, while in contrast, providers scored the item focused on privacy in the clinical setting among the highest. This apparent disconnect may be another area where both personal and culturally-driven values come into conflict with providers’ professional role. In Jordan, parents are often deeply involved in their children’s lives and there is an emphasis on the centrality of a strong family unit [34, 35]. In light of these values, our results highlight a possible conflict between cultural interpretations of the concept of confidentiality versus global definitions when it comes to informing parents. In the global literature, confidentiality is often defined as “the duty of those who receive private information not to disclose it without the patient’s consent;” however, in the Jordanian context, disclosing information to parents may not be viewed as disclosing it to an outside party [36]. Adding to this idea is that providers were, on average, relatively less supportive of the idea that youth should be given the same level of confidentiality when receiving SRH services as adults than they were of ensuring privacy at the facility during service delivery. The idea that privacy is valued over confidentiality may also be rooted in cultural norms. Privacy often refers to visual and auditory privacy in a service delivery setting, so that youth cannot be seen or heard during service encounters [36]. In Jordan, as in much of the Middle East, preserving one’s honor, and in turn a family’s honor is a priority, and should members of one’s community believe that an individual is engaging in socially-prohibited sexual behavior, it would likely bring shame upon a family [31], thus providers may feel increased responsibility for limiting the possibility of disclosure outside of the family unit. Privacy and confidentiality are aspects frequently measured as part of youth-friendly services [37], and future research to better understand how cultural definitions of confidentiality conflict with global definitions of confidentiality could be an important to identifying ways to improve youth-friendly services in Jordan.

According to our results, primary care physicians tend to have more supportive attitudes towards youth-friendly SRH service provision than do nurses and midwives. Other studies have also found that some providers, especially nurses, may have difficulty in differentiating their role as a medical professional from their role as a parent [33], and that they may judge clients based on their parental instinct and identity [38]. This phenomenon may also influence nurses and midwives’ attitudes towards adolescent sexual behavior as observed in the results of this study. Specifically, as more nurses’ and midwives are female, their attitudes may reflect their social upbringing and the impact of social scripts that prioritize family reputation and honor, which is often tied to female sexual conduct. Sexual abstinence and faithfulness among women are considered to be fundamental to a family’s honor in much of the Middle East. Given that nurses and midwives know the potential severity of disrupting a family’s honor, they may prefer to refrain from engaging with youth about issues related to their sexual behavior or concerns. Another possible explanation of this difference is that many physicians in Jordan have either trained or studied in other countries, thus they may have a different perspective towards issues pertaining to youth SRH than nurses and midwives, who most frequently complete their training in Jordan.

Value-reflective thinking around such ethical dilemmas in medical education and training programs that include self-awareness and introspective activities could help providers feel more comfortable in fulfilling their responsibilities as caregivers regardless of their personal beliefs [32]. Critical thinking around both cultural and moral dimensions of adolescent sexuality could be integrated in both pre-service and in-service trainings to help support providers when they encounter youth [4]. Further, it has been suggested that trainings geared to improve SRH service delivery towards youth may be more effective by focusing on attitudinal rather than medical issues [39] in settings where provider attitudes serve as an important limitation in providing high quality care. While in Jordan, all providers may benefit from such training, it could be that nurses and midwives may benefit the most from training focused on enabling them to better distinguish between their role as professionals and their own personal beliefs.

With regard to Subscale 2, our results show no evidence of variation in provider attitudes according to individual characteristics. This finding may suggest that the social and cultural norms measured by this scale are deeply entrenched across Jordanian society. As the attitudes held by the providers in this study appear to reflect the dominant norms and values found within their social and cultural context, our results point towards the potential for interventions focused on changing attitudes within communities at a population level to complement interventions focused specifically on individual providers. The results of this study build on the results of previous research in Jordan that highlights the potential for population-based interventions, as youth believe that social change at the community-level is closely linked with their ability to access improved SRH information and service availability [40]. In the Middle East, other studies recommend that enhancing and expanding culturally sensitive community-based efforts, especially those that include social and religious leaders, which are thought to be critical in changing norms to improve youth SRH by helping to create a more supportive environment [16, 41], and may ultimately serve to reinforce more youth-friendly attitudes among providers.

Among all the domains measured, service providers appear to have the most favorable attitudes in relation to the policy and clinical environment, as identified in Subscale 3. Further, our results suggest that providers who indicated that they have had previous training focused on RH exhibited significantly more supportive attitudes towards youth-friendly services within this domain than providers who did not report having received any training. This result is consistent with the results of another study that found that general training on RH services was associated with improved attitudes towards the provision of SRH services to adolescents [42], although the study did not examine the association between provider training and specific domains of youth-friendly services. Taking our results into consideration, it appears that even training on RH in general may be effective in helping to bring about improved provider attitudes towards making the policy and clinical environment more youth friendly, although more focused trainings may be needed to address specific social or cultural challenges to bring about improvement in relation to the other domains measured.

Last, our results show that there is some geographical variation in provider attitudes by governorate for Subscales 1 and 3. According to our results, providers in Amman tend to have more youth-friendly attitudes than providers in other governorates. This finding is not surprising, given that Amman is the largest urban area in Jordan and tends to be less conservative than other areas of the country. It is interesting, however, that there was no variation by governorate observed with regard to Subscale 2. This may again support the idea that norms and beliefs related to youth-friendly SRH services are fairly consistently held across the country and deeply entrenched in Jordanian society.

The results of this study should be interpreted in light of several strengths and limitations. A strength of this study is that we use a locally developed and validated tool to assess an understudied topic of regional and international importance. While an additional strength of this study is that it includes a large and diverse study population, the results should not be interpreted as being fully representative of providers in Jordan, as the second stage of sampling relied on recruiting individual providers by convenience. Further, this study only includes providers in four governorates in the Northern region of Jordan; there are contextual differences between the Northern and Southern regions of Jordan that could influence the findings. Even though confidentiality in responses were emphasized, providers may have been too embarrassed to answer all items truthfully as the items in the scale were often considered to be quite sensitive. The sensitivity around the issues related to youth SRH discussed in our study underscores the urgent need for programs in this area to support providers in becoming more comfortable engaging with youth. Another caveat to interpreting the results of the scale is that SRH services to youth are not routinely offered in Jordan. Therefore, providers’ attitudes may be based on reactions to the items without any real-word experience in providing SRH services to youth. Last, the scale we use was developed and tested in Jordan and many items may represent ideas that are most relevant to Jordan, or other Middle Eastern, settings, and it may not include all items relevant to other settings.

## Conclusions

To date, we are not aware of any studies in Jordan that have focused expressly on the service providers themselves within the domain of youth-friendly SRH services, despite there being several studies that suggest that youth in Jordan report negative experiences seeking SRH services [25, 43]. While clearly measuring providers’ attitudes related to youth-friendly SRH services is important to improving SRH services for youth, more research is needed to better understand providers’ perspectives, including the barriers they face, their biases and strengths, and their ideas for how they can be empowered in their roles.

## Data Availability

The datasets generated during and/or analyzed during the current study are not publicly available due to ethical restrictions but are available from the corresponding author on reasonable request.

## Abbreviation

SRH: Sexual and reproductive health
